# S109. SYMPTOM NETWORK MODELS OF PSYCHOSIS

**DOI:** 10.1093/schbul/sby018.896

**Published:** 2018-04-01

**Authors:** Adela-Maria Isvoranu

**Affiliations:** University of Amsterdam

## Abstract

**Background:**

Disorders within the psychosis spectrum are highly heterogeneous and multifactorial (Weinberger & Harrison, 2010). However, in spite of decades of research, causes of psychosis are still uncertain (e.g., Tandon et al., 2008). In an attempt to overcome these shortcomings, recent years have seen a rise in the modeling of psychotic disorders as networks of interacting symptoms (Borsboom, 2017). The centerpiece of network modeling lies in the idea that symptoms are active causal agents in producing disorder states, and that the study of their causal interaction is central to progress in understanding and treating mental disorders (Isvoranu et al., submitted). This presentation aims to introduce the network approach to mental disorders in the context of psychotic symptomatology.

**Methods:**

The network approach is a novel psychometric framework based on a dynamical systems perspective. In network models, mental disorders such as schizophrenia are no longer conceptualized as common causes of symptoms, but as conditions that arise from the interaction between symptoms. The pattern on interactions can be visualized in a network structure, in which variables (e.g., symptoms, environmental factors, genetic factors) are represented as nodes and the presence of an edge between any two nodes implies the existence of a statistical association, which does not vanish upon controlling for all of the other nodes in the network (Isvoranu et al., 2016). This talk will include two examples of network models. First, using general population data a network model for the relation between three environmental risk factors (cannabis use, developmental trauma, and urban environment), dimensional measures of psychopathology and a composite measure of psychosis is constructed (Isvoranu et al., 2016). Second, using the GROUP dataset (Korver et al., 2012) which includes patients, siblings of patients, parents and controls, a network model is constructed for the relation between a polygenic risk score for psychosis liability and symptoms of psychotic disorders.

**Results:**

The results of the first study indicate specific paths between environmental factors and symptoms, most often involving cannabis use (Isvoranu et al., 2016). In addition, the analysis suggests that symptom networks are more strongly connected for people exposed to environmental risk factors, indicating that environmental exposure may lead to less resilient symptom networks. The second study indicates that genetic vulnerability assessed via a polygenic risk score is associated with several individual psychotic symptoms – especially positive psychotic symptoms – suggesting that part of the missing heritability problem may be lie in the psychometric conceptualization of psychosis.

**Discussion:**

Psychotic disorders feature a multitude of symptoms and problems, which lead to an inherent heterogeneity of psychosis. Current (psychometric) conceptualizations of pathology cannot fully encompass the complexity of these problems – this yields to the need of developing tools that could aid our understanding of psychiatric disorders and could ultimately be implemented in clinical practice. Network modeling may provide such a tool. It is unlikely that there is such a thing as “one-size fits all treatment” for psychosis spectrum disorders, and intervention planning may require personalized network modelling (Isvoranu et al., submitted). In the coming years we are likely to learn the extent to which the network approach could aid research and clinicians.

